# Is ultra-violet radiation the main force shaping molecular evolution of varicella-zoster virus?

**DOI:** 10.1186/1743-422X-8-370

**Published:** 2011-07-27

**Authors:** Gilberto Vaughan, Araceli Rodríguez-Castillo, Mayra Y Cruz-Rivera, Karina Ruiz-Tovar, José E Ramírez-González, Pilar Rivera-Osorio, Salvador Fonseca-Coronado, Juan C Carpio-Pedroza, Fernando Cazares, Mauricio Vazquez-Pichardo, Luis Anaya, Alejandro Escobar-Gutiérrez

**Affiliations:** 1 Departamento de Investigaciones Inmunológicas, Instituto de Diagnóstico y Referencia Epidemiológicos, Secretaría de Salud, México City, México; 2 Departamento de Genoma de Patógenos, Instituto de Diagnóstico y Referencia Epidemiológicos, Secretaría de Salud, México City, México; 3 Departamento de Microbiología y Parasitología, Facultad de Medicina, Universidad Nacional Autónoma de México. México; 4 Unidad de Investigación Multidisciplinaria, Facultad de Estudios Superiores Cuautitlán, Universidad Nacional Autónoma de México. Estado de México, México; 5 Secretaría de Salud del estado de México, Estado de México, México

**Keywords:** Varicella, Ultra-violet radiation, Mexico

## Abstract

**Background:**

Varicella (chickenpox) exhibits a characteristic epidemiological pattern which is associated with climate. In general, primary infections in tropical regions are comparatively less frequent among children than in temperate regions. This peculiarity regarding varicella-zoster virus (VZV) infection among certain age groups in tropical regions results in increased susceptibility during adulthood in these regions. Moreover, this disease shows a cyclic behavior in which the number of cases increases significantly during winter and spring. This observation further supports the participation of environmental factors in global epidemiology of chickenpox. However, the underlying mechanisms responsible for this distinctive disease behavior are not understood completely. In a recent publication, Philip S. Rice has put forward an interesting hypothesis suggesting that ultra-violet (UV) radiation is the major environmental factor driving the molecular evolution of VZV.

**Discussion:**

While we welcomed the attempt to explain the mechanisms controlling VZV transmission and distribution, we argue that Rice's hypothesis takes lightly the circulation of the so called "temperate VZV genotypes" in tropical regions and, to certain degree, overlooks the predominance of such lineages in certain non-temperate areas. Here, we further discuss and present new information about the overwhelming dominance of temperate VZV genotypes in Mexico regardless of geographical location and climate.

**Summary:**

UV radiation does not satisfactorily explain the distribution of VZV genotypes in different tropical and temperate regions of Mexico. Additionally, the cyclic behavior of varicella does not shown significant differences between regions with different climates in the country. More studies should be conducted to identify the factors directly involved in viral spreading. A better understanding of the modes of transmissions exploited by VZV and their effect on viral fitness is likely to facilitate the implementation of preventive measures for disease control.

## Background

Varicella (chickenpox) follows a seasonal pattern exhibiting a higher number of primary infections occurring during spring and winter [1]. Contrary to the characteristic epidemiology of temperate regions, where varicella-zoster virus (VZV) infection is acquired early in life, in tropical regions infections among children are comparatively less common and disease is more frequent in adolescents and young adults [1,2]. Likewise, molecular epidemiology of VZV varies geographically and displays a distinctive genotype distribution, suggesting that predominance of certain genotypes in specific geographical regions is multifactorial and depends on environmental, host and viral factors [3-5]. These striking differences in the epidemiology of varicella in regard to climate and geographical location are one of the most outstanding characteristics of chickenpox [6,7].

Although several environmental factors have been suggested to play role in the distinctive epidemiological of varicella, the dramatic differences in the patterns of infection among children in the tropics remained to be explained. Recently, Rice has put forward an interesting, yet questionable, hypothesis stating that UV radiation is responsible for the geographical differences seen in epidemiology of varicella worldwide [8]. In his paper, Rice proposes that UV radiation is the main force driving the molecular evolution of VZV, initiated with the departure of man from Africa, resulting in different degrees of adaptation of viral lineages to UV radiation which has, in turn, given origin to what he refers as "temperate and tropical VZV genotypes". Moreover, the cyclic behavior of varicella is, to some extent, explained by the higher levels of UV radiation commonly observed in temperate regions during summer which are considerably less pronounced in tropical places. Although we cannot, and do not intent to, rule out completely the participation of UV radiation as an important factor affecting epidemiology of varicella, we strongly doubt that this is the main force driving VZV molecular evolution. Here, we further discuss and present novel information regarding the patterns of varicella in both, temperate and tropical regions in Mexico, along with VZV genotype distribution in the entire country. The overwhelming dominance of temperate genotypes regardless of climate in conjunction with the undoubtedly similarities in seasonal patterns between distinctive geographical locations contradict Rice's hypothesis and suggest that other still unidentified factors, rather than UV radiation, play a major role in disease pattern and dictate the distribution of VZV genotypes globally.

## Discussion

The major weakness of Rice's hypothesis is neglecting the predominance of the temperate genotypes in tropical regions. As pointed out by the author, temperate genotypes do circulate, and abundantly in some instances, in several tropical regions including Brazil, Congo, Mexico and Thailand [9-12]. While several possible explanations, including pollution, biomass burning and limited UV radiation exposure, are provided to justify the presence of non-tropical genotypes in such regions, the hypothesis bluntly fails to take into consideration the overwhelming supremacy of temperate genotypes in these regions of the world. Recently, we have reported the major genotypes circulating in Mexico City [10,11]. In conjunction, the two studies published by our group compiled a hundred twenty different isolates of European origin, belonging to either clade 1 or 3, and seven isolates corresponding to clades 5 and VI. As shown in these reports, temperate genotypes clearly dominate the region and eclipsed the minor genotypes. In Rice's paper, the overwhelming prevalence of European lineages in Mexico City is justified by the elevated levels of pollution found in the metropolitan area that supposedly reduce UV radiation intensity. Indeed, Mexico City is a highly polluted metropolis. However, according to the Atmospheric Monitoring System, UV radiation is not significantly affected http://www.calidadaire.df.gob.mx/calidadaire/index.php?opcion=2&opcioninfoproductos=23. Thus, Rice's assumption that UV radiation levels in Mexico are decreased by pollution, and therefore responsible for the striking dominance of European viruses, is baseless. Moreover, our group has been involved in a nationwide project looking at the global distribution of VZV genotypes in the country. Our preliminary data shows, unequivocally, that regardless of climate the most prevalent genotype in the entire country is European (unpublished data). There are no other metropolises in Mexico that can remotely compare to, both the territorial and pollution, extension of Mexico City. Thus, pollution should not play a major role in the vast majority of locations in the country besides Mexico City and some few other large cities (Monterrey, Guadalajara, etc.). If UV radiation was to be the major force driving VZV genotype distribution, one might expect to see a radical change in the molecular epidemiology of those regions with tropical climates. As mentioned above, we do not observe differences on the viral genotype distribution throughout the country. On the contrary, in certain tropical regions we have not been able to detect tropical genotypes, which, in general, remained fairly sporadic in Mexico.

**Figure 1:**
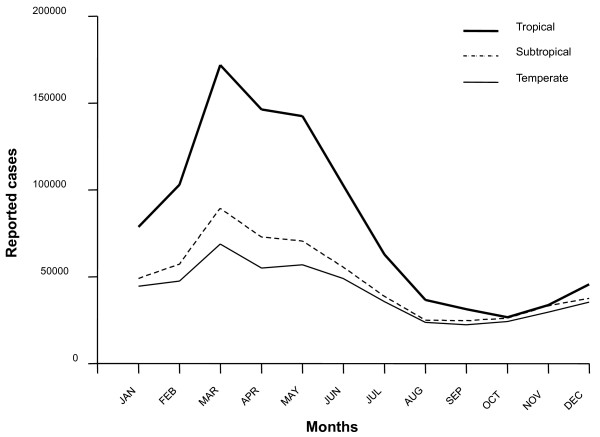
**Cyclic behavior of varicella**. The total numbers of varicella cases reported by CENAVECE during 2010 are presented. In Mexico, varicella occurs in a seasonal fashion, resulting in an increased number of infections during spring and winter beginning from mid November and quickly decreasing around mid May.

We actually accept that VZV genotype distribution follows a rather peculiar pattern but we also found this behavior difficult to be explained by the level of exposition to UV radiation alone or predominantly. Moreover, we gathered all the information regarding varicella cases reported in Mexico by the National Center of Epidemiological Surveillance and Disease Control (CENAVECE) during 2010 and separated the individual cases by regions (temperate, subtropical and tropical). Our analysis revealed the same cyclic behavior characteristic of VZV infections in a seasonal fashion which increases during spring and winter beginning from mid November and rapidly declining around mid May regardless of climate (Figure 1). As proposed by Rice, one might assume that this seasonal pattern will not be as prominent, or followed at all, in tropical and maybe subtropical regions. However, no differences in the profiles were seen, apart from the total number of cases which are higher in tropical locations. This is easily explained since in Mexico, in general terms, more individuals live in tropical regions than in temperate areas.

We believe that Rice's hypothesis is highly biased by the results published in one large study describing the global molecular epidemiology of varicella in twenty seven different countries [13]. Although the aforementioned report should be considered as a cornerstone in the study of varicella molecular epidemiology, the limited number of isolates from some regions significantly biased the sampling. Before the studies conducted in Mexico by us, the circulation of European strains in the country had not been reported and the consensus assumption indicated that clade 4 was the predominant genotype. The main shortcoming in that report regarding VZV distribution in Mexico is the number isolates collected in the country, only five, which origin was not clearly stipulated. Thus, the exact location of those isolates is unknown and whether or not those isolates are autochthonous of Mexico or imported cases is up for debate. Therefore, larger studies including representative numbers of specimens collected from tropical, subtropical and temperate regions are required to fully understand the global molecular epidemiology of VZV. The advent of the universal genotyping scheme will, most probably, favor the exchange of comparable information about viral genotype distribution around the world [14]. With these new clear guidelines regarding VZV classification and the arrival of new sequencing technologies, a wealth of information is expected to be generated in the very near future.

## Conclusion

Working hypotheses are needed, and we fully encourage their proposal, to stimulate research that can help clarify the mechanisms involved in VZV transmission. We appreciate the effort made by Philip Rice to address an interesting question on how varicella is affected by environmental factors. However, we also consider extremely important to sustain such claims with solid information supporting those facts. In our opinion, UV radiation does not satisfactorily explain the distribution of VZV genotypes in different tropical and temperate regions in Mexico and, therefore, cannot be the main force shaping evolution of VZV. Furthermore, we believe that the so called temperate genotypes are more frequent in tropical regions than suspected. Additionally, the cyclic behavior of varicella, as shown by a seasonal fashion, does not exhibit significant differences between tropical and temperate regions throughout the country, as one might expect if UV radiation was one of the major factors controlling occurrence of cases. Thus, more comprehensive studies on VZV molecular epidemiology should be conducted to identify the factors directly involved in viral spreading. A better understanding of the modes of transmissions exploited by VZV and their effect on viral fitness is likely to facilitate the implementation of preventive measures for disease control.

## Competing interests

The authors declare that they have no competing interests.

## Authors' contributions

GV, KRT and AE designed the study, analyze the data and wrote paper. All authors read and approved the final manuscript.
